# Supplementary material for: Deletion of wfs1 Impairs Oligodendrocyte Precursor Cells Dorsal Distribution and Myelination Through the wfs1-hmgcs1 Axis in Zebrafish
Source: Biology (Basel). 2026 Aug 16;15(16):1402. doi: 10.3390/biology15161402 (PMC13510151; doi:10.3390/biology15161402)
Supplement: Supplementary file 1 [file biology-15-01402-s001.zip › File S1-Transcriptome sequencing data and differentially expressed genes (DEGs) in wfs1-- zebrafish at 5 dpf/2.KEGG/wfs1KOvsWT/ALL/src/dre03018.html]

dre03018


- dre:394064

- Up regulated genes

394064\_1(5.66870555038609)

- dre:317641

- Up regulated genes

317641\_1(2.5346447835961)

- dre:448949

- Up regulated genes

448949\_1(1.74045904764039)

- Down regulated genes

448949(-1.15683913516418)

- dre:553665

- Up regulated genes

553665\_1(1.84680105357144)

- Down regulated genes

553665(-1.47971352380159)

- dre:566017

- Up regulated genes

566017\_1(4.3237862720927)

- Down regulated genes

566017(-1.85661913518901)

- dre:565659

- Up regulated genes

565659\_1(4.49651212605795)

- Down regulated genes

565659(-1.14717516835564)

- dre:373085

- Up regulated genes

373085\_1(5.15643695643451)

- dre:100538016

- Up regulated genes

100538016(1.59465916832705)
- dre:492479

- Up regulated genes

492479\_1(4.84570558850732)

- dre:321035

- Up regulated genes

321035\_1(2.36627873946462)

- dre:568001

- Up regulated genes

568001\_3(1.40501062189015)

- Down regulated genes

568001\_2(-2.92834331323733)
- dre:561416

- Up regulated genes

561416\_1(3.15253579212291)

- Down regulated genes

561416(-4.80431336765849)

- dre:324574

- Up regulated genes

565000(8.08360464769611)

- Down regulated genes

324574(-1.80226689040574)

Close
